# Chronic Losartan Treatment Up-Regulates AT_1_R and Increases the Heart Vulnerability to Acute Onset of Ischemia and Reperfusion Injury in Male Rats

**DOI:** 10.1371/journal.pone.0132712

**Published:** 2015-07-13

**Authors:** Minwoo A. Song, Chiranjib Dasgupta, Lubo Zhang

**Affiliations:** Center for Perinatal Biology, Division of Pharmacology, Department of Basic Sciences, Loma Linda University School of Medicine, Loma Linda, CA, United States of America; Indiana University School of Medicine, UNITED STATES

## Abstract

Inhibition of angiotensin II type 1 receptor (AT_1_R) is an important therapy in the management of hypertension, particularly in the immediate post-myocardial infarction period. Yet, the role of AT_1_R in the acute onset of myocardial ischemia and reperfusion injury still remains controversial. Thus, the present study determined the effects of chronic losartan treatment on heart ischemia and reperfusion injury in rats. Losartan (10 mg/kg/day) was administered to six-month-old male rats *via* an osmotic pump for 14 days and hearts were then isolated and were subjected to ischemia and reperfusion injury in a Langendorff preparation. Losartan significantly decreased mean arterial blood pressure. However, heart weight, left ventricle to body weight ratio and baseline cardiac function were not significantly altered by the losartan treatment. Of interest, chronic *in vivo* losartan treatment significantly increased ischemia-induced myocardial injury and decreased post-ischemic recovery of left ventricular function. This was associated with significant increases in AT_1_R and PKCδ expression in the left ventricle. In contrast, AT_2_R and PKCε were not altered. Furthermore, losartan treatment significantly increased microRNA (miR)-1, -15b, -92a, -133a, -133b, -210, and -499 expression but decreased miR-21 in the left ventricle. Of importance, addition of losartan to isolated heart preparations blocked the effect of increased ischemic-injury induced by *in vivo* chronic losartan treatment. The results demonstrate that chronic losartan treatment up-regulates AT_1_R/PKCδ and alters miR expression patterns in the heart, leading to increased cardiac vulnerability to ischemia and reperfusion injury.

## Introduction

Heart disease is the leading cause of morbidity and mortality in the United States. In addition to other risk factors, clinical and animal studies have shown an association between angiotensin II receptor (ATR) expression on cardiomyocytes and increased risk of ischemic heart disease and reduced cardiac recovery after ischemic injury [1–5]. Myocardial ischemia is a common form of cardiopathology. The heart may experience prolonged ischemia under a variety of conditions, including cardiomyopathy, endothelial dysfunction and coronary arterial disease, valvular dysfunction and hypotension. Animal studies suggest a link between pre-conditioning the heart with angiotensin II type 1 receptor (AT_1_R) blockers and cardiac protection in ischemia and reperfusion (IR) injury [6–8]. AT_1_R is predominately found in the adult heart and its expression is up-regulated after IR injury [1]. The AT_2_R is predominately found in the fetal and neonatal heart and its expression declines as the heart matures. AT_1_R plays an important role in the regulation of blood pressure, fluid, electrolyte balance, and is involved in pathological conditions such as cardiac remodeling and inflammation [5]. While the effect of AT_2_R is considered to be the opposite of AT_1_R, the role of AT_2_R in the heart is less clear. However, one study indicates AT_2_R is a direct antagonist by binding to AT_1_R forming heterodimerization [9].

Angiotensin II is the main activator of AT_1_R and AT_2_R. The systemic renin-angiotensin system (RAS) plays an important role in the regulation of angiotensin II levels in the circulation. In addition, the local and tissue RAS also contributes significantly to the angiotensin II production and function [10, 11]. In the heart, the local production of angiotensin II by the cardiac RAS, as well as other alternative pathways, plays a key role in the maintenance of an appropriate cellular milieu balancing stimuli inducing and inhibiting cell growth and proliferation, as well as mediating adaptive responses to myocardial stress, for example, after myocardial ischemic injury [10].

Studies in rats have shown that ischemia leads to dysregulation of ATRs in the heart and that acute pretreatment with AT_1_R blockers prior to ischemia may lead to both cardioprotective and deleterious effects [3, 8, 12, 13]. In one study, acute AT_1_R blockade impaired post-ischemic left ventricular recovery and increased AT_1_R mRNA, but did not change AT_1_R or AT_2_R protein levels [13]. However, in an *in vivo* study of acute IR, AT_1_R blockade provided improved recovery of left ventricular function, which was dependent on AT_2_R [14]. These data suggest that the outcome of cardiac recovery in IR injury is dependent on the expression levels and activity of AT_1_R and AT_2_R. The expression profiles of AT_1_R and AT_2_R are, in turn, influenced by the time and duration of AT_1_R blockade as well as IR injury [15, 16].

Thus, the effects of AT_1_R on the acute onset of myocardial ischemia and reperfusion injury and the recovery of ventricular function immediately after ischemic injury in the heart remain controversial, depending on systemic *vs*. local blockade, as well as chronic *vs*. acute blockade of AT_1_R. The present study addresses the question of whether chronic *in vivo* inhibition of AT_1_R by losartan modulates cardiac AT_1_R and AT_2_R expression, cardiac vulnerability to the acute onset of myocardial ischemia and reperfusion injury, and the recovery of ventricular function after ischemic injury in male rats. Because protein kinase C delta (PKCδ) and protein kinase C epsilon (PKCε) play pivotal roles in the regulation of myocardial ischemic injury and the mechanisms for increased heart susceptibility to IR injury involve the up-regulation of PKCδ and down-regulation of PKCε expression in the heart as demonstrated in previous studies, [17–19] we investigated the effects of AT_1_R blockade on PKCδ and PKCε expression in the left ventricle. In addition, given the recent findings that altered microRNA (miR) expression profiles are involved in cardiovascular disease including ischemic heart disease,[20–23] and that studies have shown the implication of dysregulation of miR-1, -15a-5p, -15b, -21, -24, -92a, 133a, -133b, -210, -214, -320, and -499 in the development of cardiopathology including arrhythmia, cardiac remodeling, angiogenesis, and regulation of cardiomyocyte survival [24–26]. The present study also determined the effects of chronic *in vivo* inhibition of AT_1_R by losartan on miR expression profiles in the heart. Herein, we present evidence in male rats that chronic AT_1_R blockade up-regulates AT_1_R/PKCδ and alters miR expression profiles in the left ventricle, leading to increased cardiac vulnerability to acute onset of myocardial ischemia and reperfusion injury and decreased recovery of left ventricular function immediately after ischemic injury.

## Materials and methods

### Experimental animals

Six month old male Sprague-Dawley rats were purchased from Charles River Laboratories (Portage, MI). There were two experimental protocols. In protocol 1, animals were randomly divided into two groups: saline control and losartan (Sigma, 10 mg/kg/day) treatment, continuously administered by osmotic pumps (Alzet model 2ML4, Durect Co) at 60 μl/day for 14 days. The hearts were then isolated and treated with ischemia and reperfusion (IR) in the absence of losartan in the perfusate. In protocol 2, animals were treated with saline or losartan for 14 days, and the hearts were treated with IR in the continuous presence of losartan (1 μmol/L) in the perfusate. Each group had 4–6 animals. Rats were anesthetized with isoflurane (5% for induction and 3% for maintenance) and osmotic pumps with saline or losartan were implanted subcutaneously between the shoulder blades. Femoral arteries were catheterized by polyethylene tubing (PE-10) to measure blood pressure (BP) during the treatment. The adequacy of anesthesia was determined by the loss of a pedal withdrawal reflex and any other reaction from the animal in response to pinching the toe, tail, or ear of the animal. After 14 days of treatment, hearts were isolated for functional studies and protein and miR measurements. All procedures and protocols used in the present study were approved by the Institutional Animal Care and Use Committee of Loma Linda University, and followed the guidelines by the National Institutes of Health Guide for the Care and Use of Laboratory Animals.

### Hearts subjected to ischemia and reperfusion

**Fig 1:**
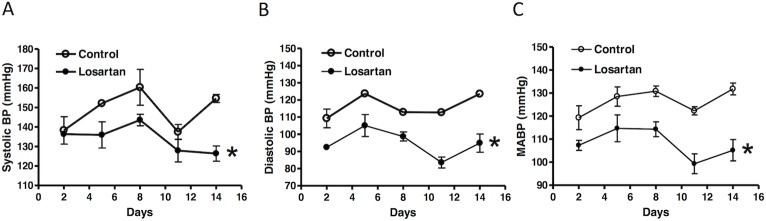
Effect of chronic losartan treatment on mean arterial blood pressure. Losartan (10 mg/kg/day) or saline were administered to male rats *via* osmotic pumps. Systolic **(A)**, diastolic **(B)**, and mean arterial blood pressure (MABP) **(C)** were measured every three days. Data were analyzed by repeated measure ANOVA. *P < 0.05, losartan *vs*. control; n = 4.

Hearts were isolated and retrogradely perfused with Krebs-Heinseleit buffer (in mmol/L: NaCl 118.5, NaHCO_3_ 25, KCl 4.7, MgSO_4_ 1.2, KH_2_PO_4_ 1.2, Glucose 11, and CaCl_2_ 2, pH 7.4) *via* the aorta in a modified Langendorff apparatus with a pressure transducer connected to a saline-filled balloon inserted into the left ventricle to assess ventricular function [18]. After the 30 minutes of baseline recording, hearts were subjected to 20 minutes of global ischemia by stopping the flow, followed by 60 minutes of reperfusion in the absence or presence of losartan (1 μmol/L) before ischemia during the baseline recording and throughout the period of ischemia and reperfusion (Fig 1). Left ventricular developed pressure (LVDP), heart rate (HR), dP/dtmax, dP/dtmin, and LV end diastolic pressure (LVEDP) were continuously recorded.

### Measurement of myocardial infarct size

Myocardial infarct size was measured as described previously [18]. In brief, at the end of reperfusion, left ventricles were collected, cut into four slices, incubated with 1% triphenyltetrazolium chloride solution for 15 minutes at 37°C, and immersed in formalin for 30 minutes. Each slice was then photographed separately, and the areas of myocardial infarction in each slice were analyzed by computerized planimetry (Image-Pro Plus), corrected for the tissue weight, summed for each heart, and expressed as a percentage of the total left ventricle weight.

### Western immunoblotting

Protein was isolated from left ventricles, and AT_1_R, AT_2_R, PKCε and PKCδ protein abundance was measured with Western blot analysis using the primary antibodies (Santa Cruz) against AT_1_R (1:1000), AT_2_R (1:2000), PKCε (1:3000) and PKC δ (1:500), as described previously [3]. Each experimental group had samples from five animals. To assure equal loading and minimize variability among gels, samples were normalized to GAPDH.

### Measurement of miRs by real-time qRT-PCR

Mature miRs were analyzed by miScript II RT kit (Qiagen) and miScript SYBR Green PCR kit with miScript Primer Assay kit (Qiagen) according to manufacturer’s instructions. Briefly, RNA was isolated from left ventricles and cDNA for mature miR profiling was prepared using the miScript II RT kit. Mature miRa were determined by real-time PCR using the miScript SYBR Green PCR kit (Qiagen). cDNA template was diluted to 1 ng/μl in RNase free water. Two nanograms of template cDNA were used for miRs quantification in a final volume of 25 μl system containing specific primers and QuantiTect SYBR Green PCR master mix following manufacturer’s instructions. Primers included miScript Universal Primer, rat-specific miScript mature miRNA primers and SNORD61 miScript Primer Assay (Qiagen). Serial dilutions of the positive control were done on each plate to create a standard curve for the quantification. The real-time PCR was performed in triplicate and threshold cycle numbers were averaged for each sample using IQ5 real-time PCR (BioRad). The expression levels of each mature miR in control and losartan-treated heart tissues were computed following the method described by Livak and Schmittgen,[27] and expressed as fold of SNORD61.

### Data analysis

Data are expressed as mean ± S.E.M. Statistical analysis (*P*<0.05) was determined by repeated measure ANOVA or Student’s *t* test, where appropriate.

## Results

### Effect of chronic in vivo losartan treatment on mean arterial blood pressure, heart rate, heart weight and heart to body weight ratio

As shown in Fig 1, losartan administration by subcutaneous osmotic implantation caused a significant decrease in systolic, diastolic and mean arterial blood pressure throughout 14 days of the treatment. There were no significant differences between saline control and losartan-treated groups in baseline heart rate (saline, 257.0 ± 63.0 *vs*. losartan, 245.0 ± 47.0, P>0.05), heart weight (saline, 1.7 ± 0.1g *vs*. losartan, 1.6 ± 0.2g, P>0.05), body weight (saline, 439.5 ± 26.2g *vs*. losartan, 465.6 ± 22.3g, P>0.05) and the left ventricle to body weight ratio (saline, 3.1 ± 0.3 mg/g *vs*. losartan, 2.9 ± 0.4 mg/g, P>0.05) after 14 days of treatment (Table 1).

**Table 1:** Effect of chronic losartan (10 mg/kg/day) treatment on heart and body weight and pre-ischemic left ventricle function.

Baseline	Saline control	Losartan
**HR (b.p.m)**	257.0±63.0	245.0±47.0
**BW (g)**	439.5±26.2	465.0±22.3
**HW (g)**	1.7±0.1	1.6±0.2
**LVW (g)**	1.3±0.1	1.4±0.2
**LVW/BW (mg g** ^**-1**^ **)**	3.1±0.3	2.9±0.4
**dP/dt**_**max**_ **(mmHg s** ^**-1**^ **)**	3353.0±285.2	3063.0±410.0
**dP/dt**_**min**_ **(mmHg s** ^**-1**^ **)**	1874.0±130.2	1700.0±193.9
**LVDP (mmHg)**	96.8±21.5	99.8±8.7

HR, heart rate; BW, body weight; HW, heart weight; LVW, left ventricular weight; LV, left ventricle; LVDP, left ventricular developed pressure; Data are mean ± SEM, n = 6.

### Effect of chronic in vivo losartan treatment on heart susceptibility to acute onset of ischemic injury

**Fig 2:**
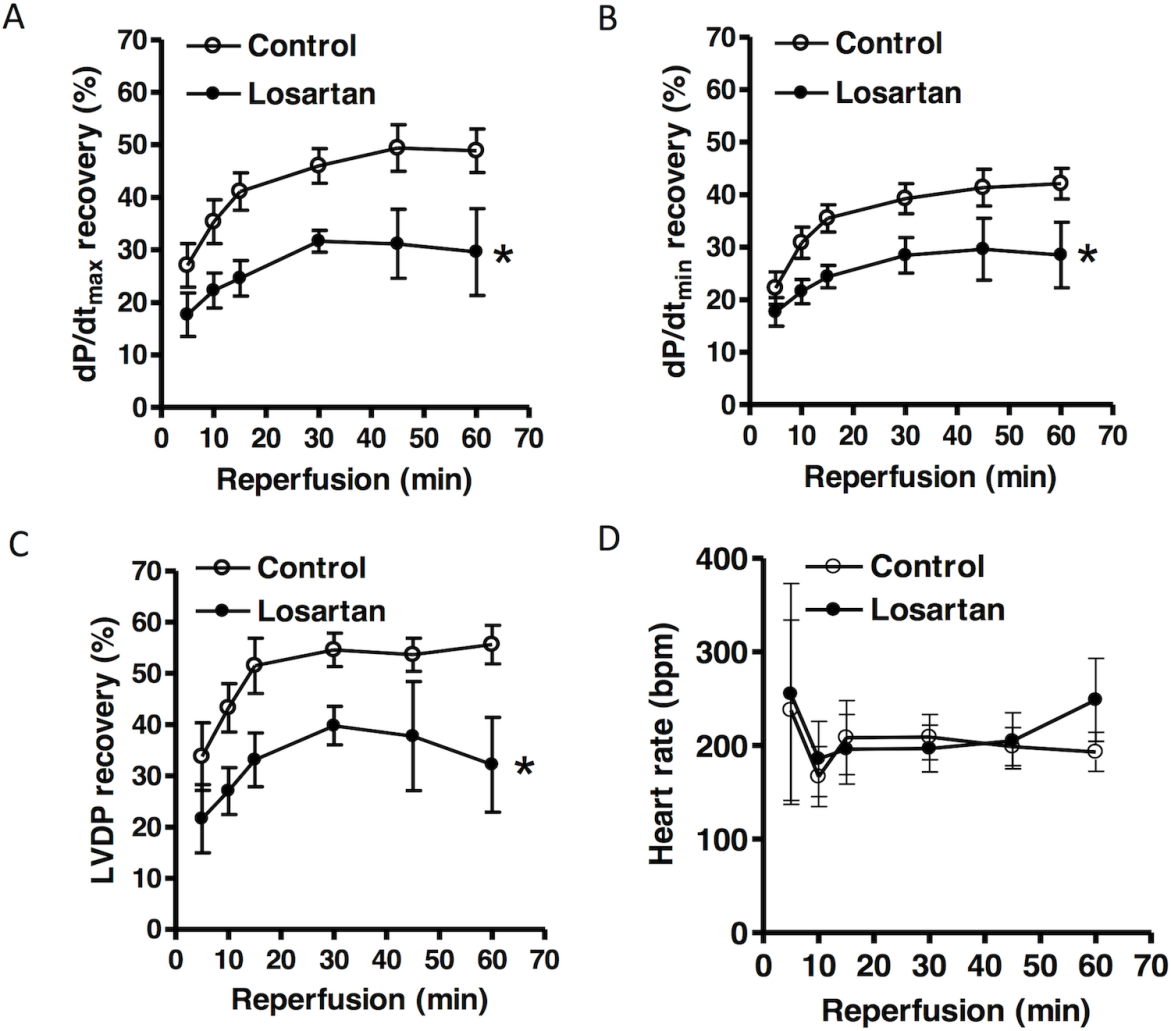
Effect of chronic losartan treatment on post-ischemic recovery of LV function. Losartan (10 mg/kg/day) or saline were administered to male rats *via* osmotic pumps for 14 days. Hearts were isolated and were subjected to 20 minutes of ischemia and 60 minutes of reperfusion in a Langendorff preparation. dP/dt_max_ **(A)**, dP/dt_min_ **(B)**, left ventricular developed pressure (LVDP) **(C)**, and heart rate (beats per minute) **(D)** were measured. Data were analyzed by repeated measure ANOVA. * *P* < 0.05, losartan *vs*. control; n = 4.

**Fig 3:**
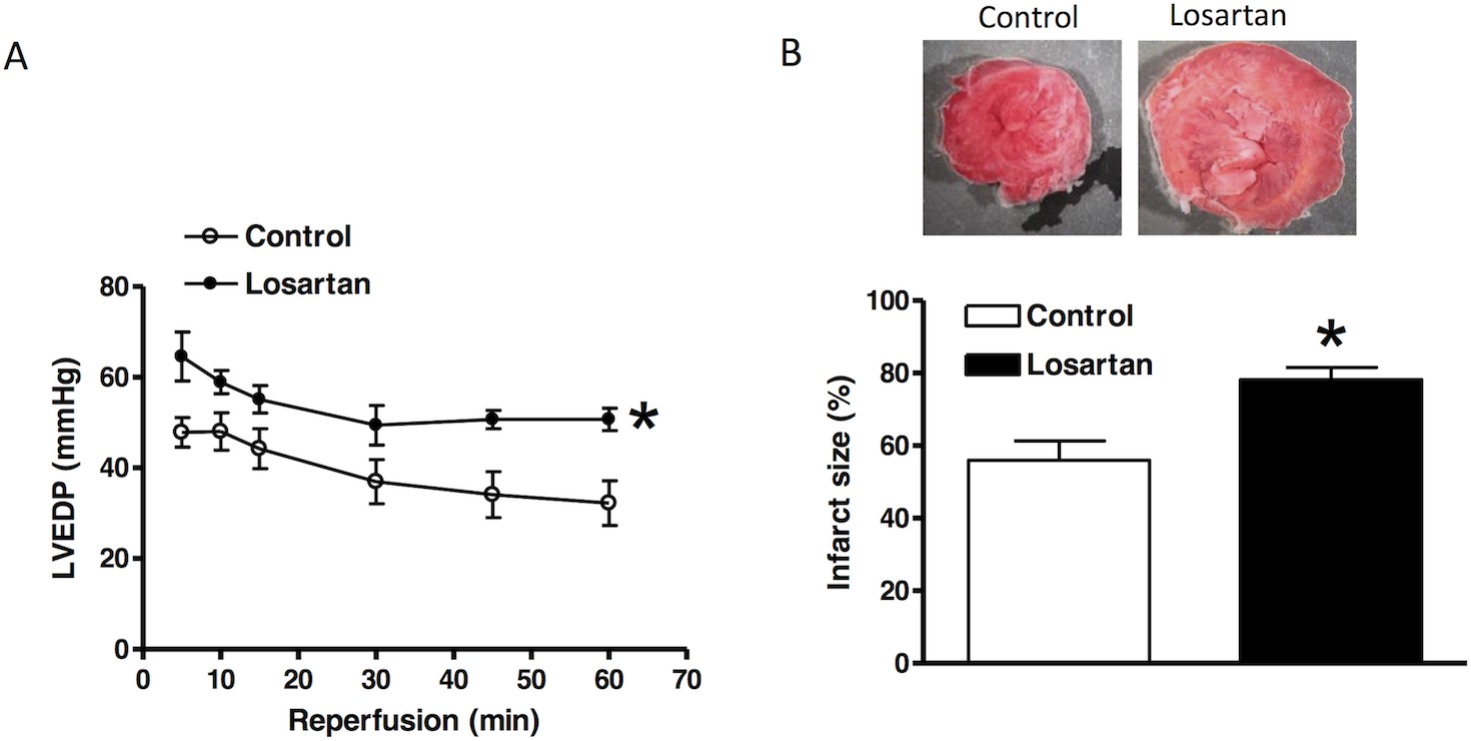
Effect of chronic losartan treatment on myocardial ischemic injury. Losartan (10 mg/kg/day) or saline were administered to male rats *via* osmotic pumps for 14 days. Hearts were isolated and were subjected to 20 minutes of ischemia and 60 minutes of reperfusion in a Langendorff preparation. Left ventricular end diastolic pressure (LVEDP) was measured during reperfusion **(A)**. Myocardial infarct size (**B**) was determined at the end of reperfusion and expressed as a percentage of the total left ventricle weight, as described in Methods. LVEDP data were analyzed by repeated measure ANOVA, and infarct size by *t*-test. * *P* < 0.05, losartan *vs*. control; n = 4.

Hearts were isolated from animals treated with saline or losartan for 14 days, and were studied in a Langendorff preparation. There were no significant differences in left ventricle developed pressure (LVDP), heart rate (HR), dP/dt_max_ and dP/dt_min_ at the baseline between the two groups (Table 1). Global ischemia for 20 minutes caused a significant impairment in LV function in both groups. As shown in Fig 2, compared with the control group, there were significant decreases in post-ischemic recovery of LVDP, dP/dt_max_ and dP/dt_min_ in the losartan treatment group. Recovery of HR was not significantly different between the control and losartan groups (Fig 2). As shown in Fig 3, global ischemia caused myocardial infarction and increased left ventricle end diastolic pressure (LVEDP). Compared with the control group, chronic *in vivo* losartan treatment significantly increased post-ischemic LVEDP and myocardial infarct size (Fig 3).

### Effect of chronic in vivo losartan treatment on AT1R, AT2R and PKC isozyme expression

**Fig 4:**
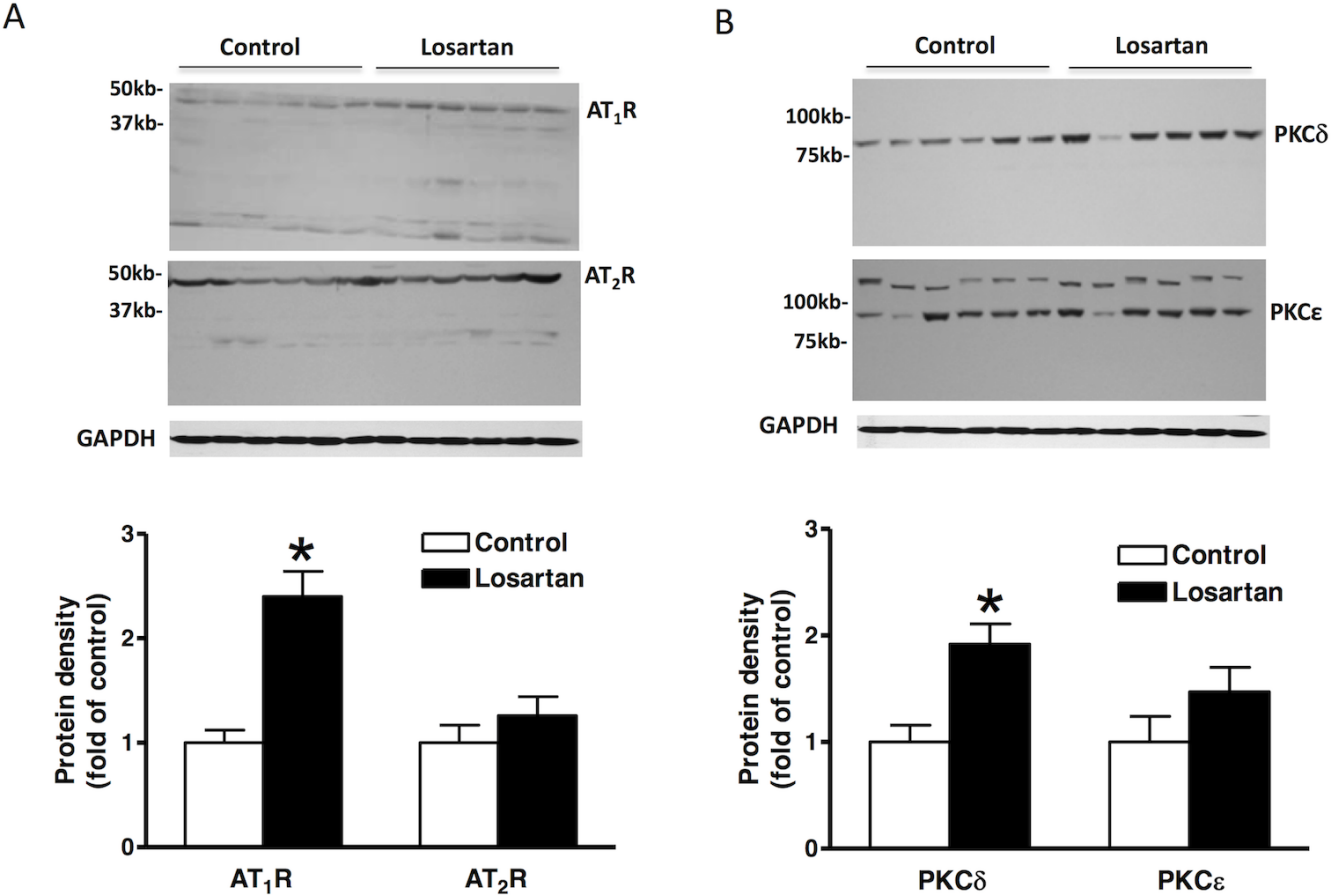
Effect of chronic losartan treatment on protein expression in the left ventricle. Losartan (10 mg/kg/day) or saline were administered to male rats *via* osmotic pumps for 14 days. Protein abundance of AT_1_R, AT_2_R (**A**), PKCδ, PKCε (**B**) in the left ventricle was determined by Western blot analyses. Data were analyzed by *t*-test. * *P* < 0.05, losartan *vs*. control; n = 6.

The protein abundance of AT_1_R, AT_2_R, PKCε and PKCδ in the left ventricle was determined by Western blot analysis. As shown in Fig 4, AT_1_R levels were significantly increased in the losartan treatment group than those in the saline control (P<0.05). Unlike AT_1_R, AT_2_R protein abundance was not significantly altered by the losartan treatment. Differential expression of PKC isozyme proteins was also identified in the left ventricle. Whereas there was no significant difference in PKCε protein levels between saline control and losartan-treated animals, the level of PKCδ was significantly greater in the losartan treatment group than those in the saline control (P<0.05) (Fig 4).

### Effect of chronic in vivo losartan treatment on miR expression profiles in the left ventricle

**Fig 5:**
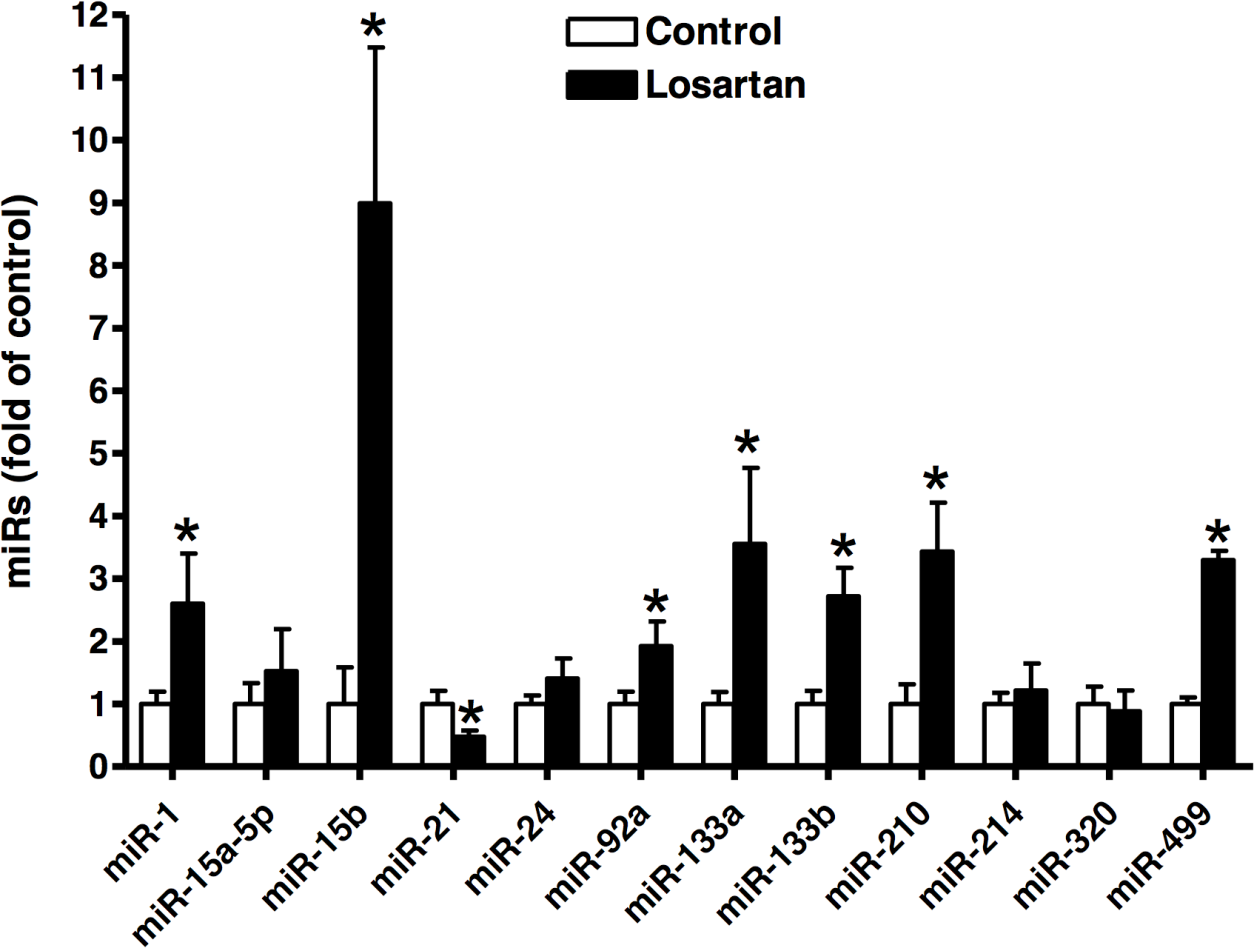
Effect of chronic losartan treatment on microRNA (miR) expression profiles in the left ventricle. Losartan (10 mg/kg/day) or saline were administered to male rats *via* osmotic pumps for 14 days. MiR expression profiles in the left ventricle was determined by qRT-PCR. Data were analyzed by *t*-test. * *P* < 0.05, losartan *vs*. control; n = 4.

Although several recent studies have reported the signature of miR expression profiles in IR-related injuries, the results have varied depending on the duration of ischemia and direct correlation between ATR activity and miR expression in the heart remains largely elusive [20, 28]. As shown in Fig 5, miR-1, -15b, -92a, -133a, -133b, -210, and -499 levels were significantly increased in the left ventricle of animals with chronic *in vivo* losartan treatment, as compared to the saline control group (P<0.05). In contrast, miR-21 expression was significantly decreased in the left ventricle (P<0.05). The losartan treatment had no significant effect on the expression of miR-15a-5p, -24, -214, and -320 in the heart.

### Effect of chronic in vivo losartan treatment on heart susceptibility to acute onset of ischemic injury in the presence of losartan during ex vivo perfusion of the heart

**Fig 6:**
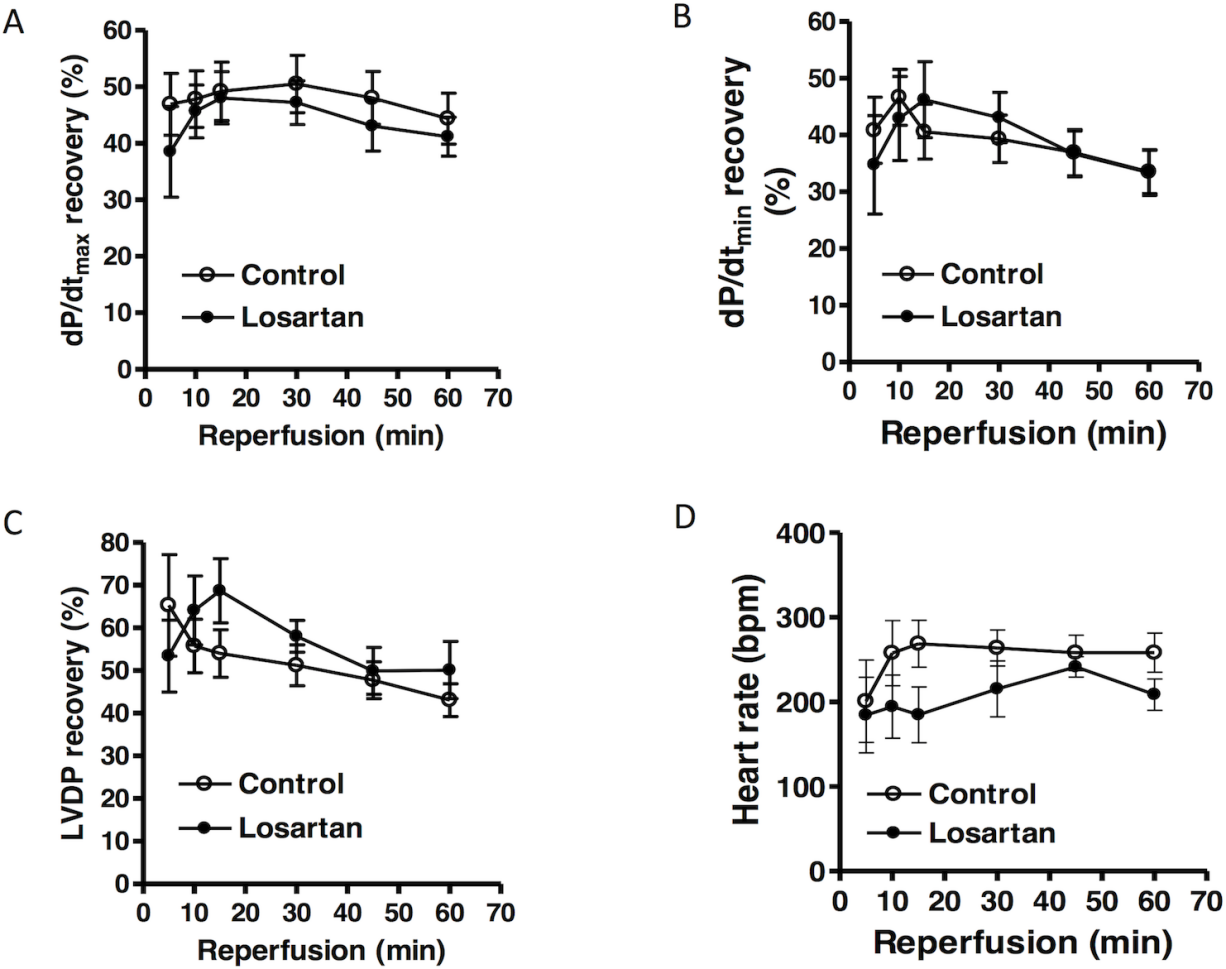
Effects of *ex vivo* AT_1_R blockade on chronic *in vivo* losartan-modulated post-ischemic recovery of LV function. Losartan (10 mg/kg/day) or saline were administered to male rats *via* osmotic pumps for 14 days. Hearts were isolated and were subjected to 20 minutes of ischemia and 60 minutes of reperfusion in a Langendorff preparation in the continued presence of losartan (1 μmol/L). dP/dt_max_ (**A**), dP/dt_min_ (**B**), Left ventricular developed pressure (LVDP) (**C**), and heart rate (beats per minute) (**D**) were determined. Data were analyzed by repeated measure ANOVA.

**Fig 7:**
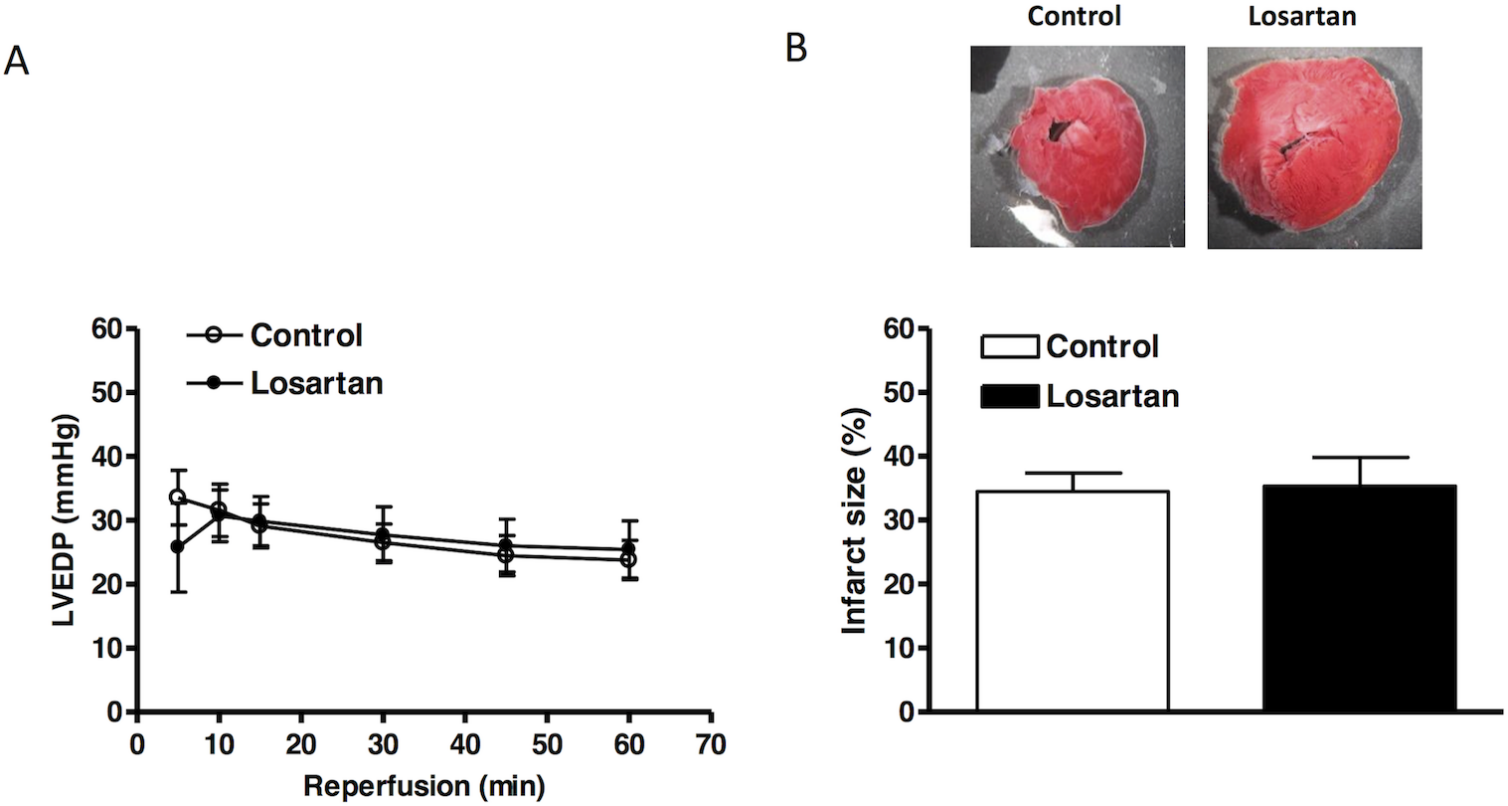
Effects of *ex vivo* AT_1_R blockade on chronic *in vivo* losartan-modulated myocardial ischemic injury. Losartan (10 mg/kg/day) or saline were administered to male rats *via* osmotic pumps for 14 days. Hearts were isolated and were subjected to 20 minutes of ischemia and 60 minutes of reperfusion in a Langendorff preparation in the continued presence of losartan (1 μmol/L). Left ventricular end diastolic pressure (LVEDP) was measured during reperfusion (**A**). Myocardial infarct size (**B**) was determined at the end of reperfusion and expressed as a percentage of the total left ventricle weight, as described in Methods. LVEDP data were analyzed by repeated measure ANOVA, and infarct size by *t*-test.

We further determined the functional significance of the *in vivo* losartan treatment-induced increase in AT_1_R in modulating heart vulnerability to acute onset of ischemic injury by blocking AT_1_R with losartan during the *ex vivo* perfusion of the heart in a Langendorff preparation. As shown in Table 2, there were no significant differences in baseline LV function between the saline control and *in vivo* chronic losartan treatment groups in the presence of losartan (1 μmol/L) during the *ex vivo* perfusion of the heart in the isolated heart preparation. Of importance, continued blockade of AT_1_R with losartan during *ex vivo* perfusion of the heart blocked the *in vivo* chronic losartan treatment-induced increase in heart susceptibility to acute onset of ischemic injury. As shown in Fig 6, in the presence of losartan during the heart perfusion in a Langendorff preparation, there were no significant differences in post-ischemic recovery of LVDP, HR, dP/dt_max_ and dP/dt_min_ between the saline control and *in vivo* chronic losartan treatment groups. Consistent with these findings, there were no significant differences in myocardial infarct size and LVEDP between the two groups (Fig 7).

**Table 2:** Effect of chronic losartan (10 mg/kg/day) treatment on heart and body weight and pre-ischemic left ventricle function in the continued presence of losartan in *ex vivo* heart perfusion.

Baseline	Saline	Losartan
HR (b.p.m)	275.0±33.0	259.0±11.0
BW (g)	470.5±25.1	445.5±13.6
HW (g)	2.0±0.1	1.8± 0.1
LVW (g)	1.5±0.1	1.3±0.0
LVW/BW (mg g^-1^)	3.1±0.3	2.9±0.4
dP/dt_max_ (mmHg s^-1^)	4367.0±83.6	4401.0±263.3
dP/dt_min_ (mmHg s^-1^)	2654.0±54.5	2511.0±108.6
LVDP (mmHg)	110.1±5.6	108.9±3.2

HR, heart rate; BW, body weight; HW, heart weight; LVW, left ventricular weight; LV, left ventricle; LVDP, left ventricular developed pressure; Data are mean ± SEM, n = 4.

## Discussion

The present study demonstrates that *in vivo* chronic AT_1_R blockade with losartan leads to a significant increase in AT_1_R and PKCδ protein expression as well as an ischemic-sensitive signature of miR expression in the left ventricle, resulting in an increase in the heart vulnerability to acute onset of ischemic and reperfusion injury and a decrease in post-ischemic left ventricular function recovery in an isolated heart preparation. In addition, this heightened heart susceptibility to acute ischemic injury induced by chronic *in vivo* losartan treatment is inhibited by the continued blockade of AT_1_R during *ex vivo* heart perfusion, suggesting a role for the increased AT_1_R expression in the left ventricle.

In studies involving *acute* losartan treatment, inhibition of AT_1_R lead to improved cardiac recovery, but the potential gain in function was abrogated by AT_2_R inhibition [14, 29]. This suggests that during AT_1_R inhibition angiotensin II concentrations increase, resulting in increased activation of the AT_2_R, conferring cardioprotection *via* production of nitric oxide [29, 30]. In the present study, chronic *in vivo* losartan treatment did not increase AT_2_R density, but increased AT_1_R expression. It has been shown that cardiac overexpression of the AT_2_R attenuates left ventricle remodeling after myocardial infarction [31]. Therefore, in addition to our findings, it is plausible that AT_2_R acts as a fail-safe switch to counteract the AT_1_R during increased levels of angiotensin II. However, there may be a limit to which AT_2_R can compensate and the increase in AT_1_R activity may overwhelm the AT_2_R ability to antagonize AT_1_R.

Previous studies with chronic antagonism of AT_1_R reported varying results. In one study, 1 and 3 weeks treatment with losartan or UP269-6, a noncompetitive AT_1_R blocker, increased AT_2_R protein, but caused no change in AT_1_R [32]. In addition, while losartan was able to preserve, but not improve, post-ischemic LV function, UP269-6, considered a more effective AT_1_R antagonist, resulted in a significant decrease in LV recovery [32]. Other studies have shown that chronic AT_1_R antagonism decreases infarct size, increases AT_1_R and AT_2_R mRNA, and increases PKCε protein, but no functional results were reported [15, 33]. Cardiac recovery following acute pretreatment of AT_1_R blockers in animal models of IR injury is variable [3, 8, 13, 34]. Thus, even subtle protocol differences may explain these variable findings. In addition, the present finding suggests that the outcome of cardiac recovery immediately after IR injury is dependent on the expression levels and activity of AT_1_R, which are also sensitive to protocol differences, *i*.*e*. blockade of AT_1_R before, during and after the ischemic insult, as well as systemic *vs*. direct local heart blockade or chronic *vs*. acute blockade of AT_1_R.

In the present study, the heart became more vulnerable to acute onset of ischemic injury after the chronic losartan treatment. While this finding is somewhat surprising and may appear contrary to the view that AT_1_R hyperactivity is implicated in ischemic injury of the heart, it indeed is likely due to the up-regulation of AT_1_R in the heart by chronic blockade of AT_1_R. Since losartan is a competitive AT_1_R blocker, *in vivo* chronic administration leads to an increase in AT_1_R abundance in the heart in a negative feedback manner. This increased AT_1_R density and activation in subsequent acute onset of ischemic insult in the isolated heart preparation was unopposed, which led to increased myocardial infarction and decreased post-ischemic recovery of left ventricular function. This notion is supported by the finding that the post-ischemic left ventricular function was preserved in *in vivo* chronic losartan treated group with continued losartan presence in *ex vivo* perfusion of the heart. Although this finding suggests that increased AT_1_R density in the heart may be detrimental to the heart in response to acute onset of ischemic injury, previous studies of losartan on the recovery of left ventricular function in the setting of ischemia and reperfusion injury in isolated rat hearts suggested a detrimental effect of the acute AT_1_R blockade [3, 12]. These findings are intriguing and suggest that the local and direct activation of cardiac AT_1_R in the regulation of heart response to acute onset of ischemic insult is much more complex than was previous thought and is likely to be context-specific. Thus, maintaining the normal level of AT_1_R activity in the heart appears important and either increased or decreased AT_1_R activation is likely to be detrimental to the heart in the setting of acute ischemia and reperfusion injury.

The finding of increased PKCδ in the left ventricle provides a mechanistic insight into the understanding of losartan-mediated up-regulation of AT_1_R and increased heart vulnerability to ischemic injury. Whereas PKCε has been implicated in cardioprotection, [35] increased PKCδ activity has coincided with a reduction in cardiac recovery in the setting of acute ischemia and reperfusion injury [36]. It has been shown that PKCδ inhibits glyceraldehyde-3-phosphate dehydrogenase and promotes the accumulation of mitochondria near lysosomal-like structures, which leads to an increase in mitochondrial permeability [37]. Several recent studies demonstrated that PKCδ played a key role on AT_1_R-mediated inflammation, oxidative stress and cardiac remodeling with cardiac fibroblasts [38–40].

In addition, the present study demonstrates that chronic *in vivo* losartan treatment alters miR expression profiles in the left ventricle with a signature of heightened cardiac vulnerability to ischemic injury. Up-regulation of miR-1, -15b, -92a, -133a, and -133b have been shown to be involved in the regulations of multitude of genes in the heart, contributing to the development of arrhythmia, hypertrophy, fibrosis, and suppression of angiogenesis, and cell death and survival [41–48]. In comparison, the elevated expression of miR-210 and miR-499 are more in favor with cell survival. MiR-210 induces angiogenesis and miR-499 stimulates cardiac stem cells to commit into mature working cardiomyocytes, [49–51] albeit cardiomyocytes are terminally differentiated in mature adult hearts and there are minimal cardiac stem cells to be stimulated. In addition, miR-1, miR-15 and miR-21 can directly influence the survival of cardiomyocytes. Both miR-1 and miR-15b target Bcl-2 down-regulation [47, 48] and thus, increase the cardiomyocyte susceptibility to apoptosis in the setting of ischemia and reperfusion injury. In contrast, miR-21 has been shown to promote cardioprotection by targeting *PDCD4* [52]. Thus, the finding that chronic losartan treatment significantly increased miR-1 and, particularly miR-15b of 9-fold, as well as decreased miR-21 by 50% in the left ventricle, suggests a novel mechanism of miRs in angiotensin receptor-modulated vulnerability of the heart in response to acute onset of ischemic injury. The question of whether this chronic losartan-induced, ischemic-sensitive signature of miR expression in the heart is caused by AT_1_R blockade or by an unopposed activation of cardiac AT_2_R remains to be determined. Future studies are needed to determine how AT_1_R interaction with PKCδ and miR mediate heart susceptibility in the presence of losartan during *ex vivo* perfusion of the heart.

In perspective of clinical significance, inhibition of AT_1_R is an important pharmacological therapy in the management of hypertension, particularly with long-term benefits in the treatment of patients in post-myocardial infarction period and at risk for ischemic heart disease. Yet the role of cardiac AT_1_R in acute onset of myocardial ischemia and reperfusion injury and left ventricular function recovery immediately after the ischemic insult still remains controversial. In our first experimental protocol, animals were pretreated with losartan or saline for two weeks duration. However, losartan was absent during the IR treatment of the heart. In clinics, losartan is used in conjunction with β-blockers and diuretics in patients with high risk for coronary artery disease and patients with prior ischemic events. The first experimental protocol was designed to replicate prolonged use of losartan followed by withdrawal from losartan, which is seen in patients transitioning to angiotensin-converting-enzyme inhibitor (ACEi), non-compliance, or reduction in risk factors. Our results demonstrated that the prolonged treatment of losartan significantly increased AT_1_R abundance in the myocardium, which was associated with increased IR-induced myocardial injury and decreased cardiac functional recovery. To determine whether this increased injury was indeed AT_1_R-mediated, in our second experimental protocol, animals were pretreated with losartan or saline for two weeks, followed by IR treatment of the heart in the continuous presence of losartan in the perfusate. The results showed that the presence of losartan during the IR treatment blocked the losartan pretreatment-induced increase in myocardial injury, providing the evidence of a causal role of AT_1_R in ischemic myocardial injury. Taken together in clinical perspective, these findings suggest that patients who are on prolonged losartan treatment significantly increase AT_1_R in the heart and activation of elevated AT_1_R in the acute setting of IR is detrimental to cardiac recovery. Therefore patients are more susceptible to ischemic heart injury during the initial withdrawal of losartan and gradual losartan withdrawal should be considered to allow sufficient time to decrease AT_1_R in the myocardium. It should be noted that the present study was conducted in animals with healthy hearts. Thus, it is unclear whether chronic losartan treatment in patients who have experienced prior myocardial ischemia and infarction elevates AT_1_R and increases heart susceptibility to IR. Given that remote ischemic preconditioning (RIPC) has clinical potential to minimize myocardial infarction in patients with high risk [53] and that RIPC mediates protection indirectly via remote humoral conditioning including adenosine, bradykinin, opioids, and HIF but minimal correlation with AT_1_R [54, 55], the present finding may not suggest the exclusion of RIPC for patients with sartans. ARBs are an important group of antihypertensive drugs, the present finding is of critical importance in the clinical perspective, and suggests a potential serious side effect of abrupt withdrawal in the ARB treatment in an increased risk of myocardial infarction in the setting of acute ischemic injury. Whereas the present study was conducted in male rats, the previous study demonstrated that the acute and direct effects of AT_1_R and AT_2_R on modulating acute ischemia and reperfusion injury in rats were in a gender-independent manner [3]. Nonetheless, the effect of chronic AT_1_R blockade on the heart sensitivity to acute onset of ischemic injury in females remains to be determined.

^1^ **Sources of Funding:** This work was supported by National Institutes of Health Grant HL118861 (LZ).

^2^ **Disclosures:** None.
